# Vitamin D and Its Metabolites Status before and during Chemotherapy in Caucasian Breast Cancer Patients

**DOI:** 10.3390/metabo13090996

**Published:** 2023-09-06

**Authors:** Małgorzata Kimsa-Furdzik, Anna Bednarek, Grzegorz Hibner, Paulina Czajka-Francuz, Sylwia Cisoń-Jurek, Dobromiła Karawasiecka, Bożena Szymczak, Jerzy Wojnar, Jerzy Chudek, Tomasz Francuz

**Affiliations:** 1 Department of Biochemistry, Faculty of Medical Sciences in Katowice, Medical University of Silesia, Medyków 18 St., 40-752 Katowice, Poland; malgorzata.kimsa@sum.edu.pl (M.K.-F.); ghibner@sum.edu.pl (G.H.); 2 1st Department of Cardiology, Medical University of Silesia, 47 Ziołowa St., 40-635 Katowice, Poland; annabednarekmd@gmail.com; 3 Department of Internal Medicine and Oncological Chemotherapy, Faculty of Medical Sciences in Katowice, Medical University of Silesia, Reymonta 8 St., 40-027 Katowice, Poland; paulinaczajka@op.pl (P.C.-F.); sylwiacison@o2.pl (S.C.-J.); dobromilak@gmail.com (D.K.); bszymczak@sum.edu.pl (B.S.); jwojnar@sum.edu.pl (J.W.); chj@poczta.fm (J.C.)

**Keywords:** vitamin D, metabolites, breast cancer, catabolism, survival, liquid chromatography/tandem mass spectrometry (LC–MS/MS)

## Abstract

Background: The predictive role of vitamin D (VD) in breast cancer (BC) patients’ survival is still being investigated. This paper aims to evaluate the changes in VD metabolites during chemotherapy (CTH) and the predictive role of VD status in Caucasian BC patients treated with CTH. Methods: Vitamin D and its metabolites were assessed with reference LC–MS/MS methodology in 98 consecutive BC patients starting CHT, after 3 and 6 months, and compared to the control group. Results: The frequency of VD deficiency in BC patients was greater than in the control group (56.1% vs. 37.2%). After 6 months of CTH, the number of VD-deficient BC patients slightly increased to 60%. The concentrations of VD active forms [25(OH)D_2_, 25(OH)D_3_], and catabolites [24,25(OH)_2_D_3_ and 3-epi-25(OH)D_3_] decreased after 3 and 6 months of CTH compared to the baseline values. Strong positive correlations between concentrations of 3-epi-25(OH)D_3_ and 25(OH)D in both groups were found. Similar correlations were also observed between 24,25(OH)_2_D_3_ and 25(OH)D levels. Kaplan–Meier survival analysis showed significantly longer survival in BC patients without deficiency (>20 ng/mL) at baseline (HR = 2.44 (95% CI 1.07–5.59), *p* = 0.026). Conclusions: (1) Our data provide further evidence that BC patients before CTH are more VD-deficient than the general population and this deficiency increases further during CTH treatment, as observed using the reference LC-MS methodology. (2) Presented results show that VD catabolism is not affected in BC patients. (3) The poorer survival in VD-deficient BP patients supports the importance of VD supplementation in BC patients with 25(OH)D levels below 20 ng/mL.

## 1. Introduction

Breast cancer (BC) is the most common malignancy and cause of cancer-related mortality among women worldwide [1]. Vitamin D (VD) via stimulation of Vitamin D receptor (VDR) plays an important role in BC development, modulating cancer cell proliferation, differentiation, apoptosis, and epithelial–mesenchymal transition, as shown in preclinical and clinical studies [2,3,4]. VD plays a critical role not only in cancer, but also in maintaining homeostasis in various conditions including cardiovascular and lung diseases, diabetes, infections and pregnancy. VD deficiency has a negative impact on all-cause mortality [5].

New research emphasizes the involvement of VD in the regulation of tumor metabolism [6]. Of note, BC cells have a similar ability to convert the 25-OH metabolite of the active form—1,25(OH)_2_ D as periurethral cells in the kidney, and this process is independent of the hydroxylation regulation in the kidneys [7].

Low levels of VD increase the risk of BC development [8], and may affect the efficacy of treatment and patients’ prognosis [7,9]. The metabolic effects of VD are mediated not only by its active form but also metabolites [10]. 

Laboratory assessments of VD status in daily clinical practice are usually limited to 25-monohydroxylated forms: 25(OH)D_2_ and 25(OH)D_3_. Contrary to the main active form, 1,25(OH)_2_D, characterized by a short half-life (of 4–6 h), monohydroxylated forms have a longer half-life of app. 15 days [11]. These compounds can be assessed with immunoassays, high-performance liquid chromatography (HPLC), or liquid chromatography/tandem mass spectrometry (LC–MS/MS). Immunoassays assessments offer high sensitivity and automation. However, this method could over- or underestimate the total 25(OH)D level depending on the immunoassay type [12,13] due to the cross-reactivity of different VD forms. HPLC is characterized by low costs, but has low sensitivity and reduced ability to distinguish VD metabolites. LC–MS/MS is more expensive, but it identifies VD metabolites with high specificity and sufficient sensitivity. This technique also enables quantification and differentiation between 25(OH)D_2_ and 25(OH)D_3_ and other VD derivatives. Thus, LC–MS/MS is considered as the current reference method for the determination of VD [14,15]. 

The majority of BC patients before [7,15], and even more after chemotherapy (CTH) [16,17,18] demonstrated VD deficiency, but few previously published studies [7,19,20,21] used LC–MS/MS assessments. Importantly, metabolically active vitamin D_2_ and D_3_ derivatives levels depend significantly on the patient’s genotype [11], but limited data regarding BC Caucasian population have been published so far. That is why we aimed to assess baseline VD status and its changes during CTH using LC–MS/MS methodology in the Caucasian population.

Vitamin D metabolic effects depend on its catabolism. Thus, we considered the significance of its catabolic pathways in BC patients. Vitamin D is catabolized mainly by the transformation of 1,25(OH)_2_D and its precursor 25(OH)D via 24-hydroxylase enzyme into 24,25(OH)_2_D and 1,24,25(OH)_3_D derivatives, known as inactive metabolites. Far less information is available about the function of the C_3_-epimerase catabolic pathway [22,23], resulting in 3-epi-25(OH)D_3_ synthesis. However, due to different impacts on calcium and bone metabolism [24], distinguishing between 25(OH)D_3_ and the C-3 epimer is considered of biological importance [25]. So far, few clinical data are available regarding 3-epi-25(OH)D_3_ in BC patients. Therefore, we assessed the level of VD catabolites, 24,25(OH)_2_D_3_ and 3-epi-25(OH)D_3_, to provide further data on VD catabolic pathways in these patients. 

Previous studies showed that VD could affect BC patients’ survival; however, the data are inconsistent [26].

We evaluated changes in VD metabolites during CTH and the predictive role of VD status in Caucasian patients with BC treated using CTH via the LC–MS/MS methodology.

## 2. Materials and Methods

### 2.1. Subjects

All patients screened for the study were admitted into the Department of Internal Medicine and Chemotherapy between 2013 and 2018. The main inclusion criteria included the diagnosis of BC confirmed with pathology result: Eastern Cooperative Oncology Group (ECOG) performance status ≤ 2. The main exclusion criteria were pregnancy or lactation, acute or chronic inflammatory diseases, and Vitamin D supplementation. All patients signed an informed consent form before enrollment. Finally, 105 women with BC were enrolled in the study. Subjects received CTH as neoadjuvant, adjuvant, or palliative treatment with or without surgery, as planned by the oncologist, in accordance with the current clinical guidelines of the National Comprehensive Cancer Network (NCCN), dated 2014 [27]. Patients were assessed at baseline and followed-up for 6 months after the start of treatment, then assessed for survival. Vitamin D levels were compared to the control group which consisted of 43 all-comer women [age 59.0 ± 17.5 years; BMI 28.0 (24.9–29.7) kg/m^2^; mean values ± SD] admitted consecutively into the endocrinology ward for the diagnosis of suprarenal gland adenoma, found free of the disease. Characteristics of the study group are shown in Table 1. The study was approved by the Bioethics Committee of the Medical University of Silesia in Katowice (EC approval no: KNW/0022/KB1/2/15) and was conducted in accordance with the Declaration of Helsinki. 

### 2.2. Materials

Metabolites of Vitamin D (25(OH)D_3_, 3-epi-25(OH)D_3_, 25(OH)D_2_, and 24,25(OH)_2_D_3_) and deuterated internal standards (*d_6_*-25(OH)D_3_, *d_3_*-3-epi-25(OH)D_3_, *d_3_*-25(OH)D_2_, and *d_6_*-24,25(OH)_2_D_3_) were purchased from Sigma-Aldrich (Gillingham, Dorset, UK). During sample preparation, several reagents were used, such as water, methanol, ethyl acetate, hexane (Honeywell, Sigma-Aldrich), and zinc sulfate (POCh S.A., Gliwice, Poland). 4-(4′-dimethylaminophenyl)-1,2,4-triazoline-3,5-dione (DAPTAD) was used as a derivatization agent. It was synthesized by Masdiag Laboratory (Warsaw, Poland). For chromatographic separation, Agilent Eclipse ZORBAX XDB-C18 (1.7 m; 50 × 4.6 mm) column was used. (Advanced Chromatography Technologies Ltd., Aberdeen, Scotland). 

### 2.3. Apparatus and Chromatographic Conditions

ExionLC high-performance liquid chromatograph (Sciex, Framingham, MA, USA) with CTC PAL autosampler (Zwinger, Switzerland) coupled with QTRAP^®^ 4500 MS/MS system (Sciex) was used. Liquid chromatograph was equipped with a degasser, two pumps, and a column oven. Analyses were performed in a positive mode using electrospray ionization (ESI). For quantitative analysis, multiple reaction monitoring (MRM) was used. The ion source parameters were optimized with the flow injection analyses of the standards mixture. The following operating parameters of MS/MS system were applied: curtain gas (CUR) 30, ion source voltage (IS) 3000 V, temperature (TEM) 500 °C, nebulizing gas (GS1) 40, and drying gas (GS2) 50. CUR, GS1, and GS2 values are expressed in arbitrary units. The raw data were collected with the use of Analyst Software. Multiquant Software was used to process and quantify the collected data. The chromatographic analysis was performed using the Agilent Eclipse ZORBAX XDB-C18 (1.7 m; 50 × 4.6 mm) column at a flow rate of 0.8 mL∙min^−1^ and the column oven temperature was 40 °C. The mobile phase was prepared using water and acetonitrile with 0.1% formic acid. The gradient elution program was as follows: 0 min.—50% B, 2.5 min.—78% B, 3.2 min.—98% B, 4.5 min.—98% B, and 4.6 min.—50% B. The injection volume was 20 μL. Total run time was 5.5 min. The quantitative analysis of vitamin D metabolites was performed using the isotope dilution method. The concentration was calculated on the basis of the ratio of the area of a given metabolite peak to the area of the internal standard peak. The obtained values from serum were compared with the calibration curve. All LC-MS/MS assessments were performed according to the methodology described in detail in the publications [28,29].

### 2.4. Serum Sample Preparation

Venous blood samples were collected before the administration of CTH and during the selected follow-up visits (after 3 and 6 months of CTH initiation). Vitamin D concentration was assessed in serum samples stored at a temperature below −170 °C until analysis was carried out. The sample preparation process was started by incubating 100 µL of serum with 10 µL of solution of isotope-labeled standards for 30 min. Then, protein precipitation was performed using 0.2 M zinc sulfate solution and methanol. The mixture was vortexed for 10 s. Subsequently, liquid–liquid extraction was carried out using hexane as an extractant. The extraction was performed twice. After each extraction step, the sample was vortexed and centrifuged (13,000 RPM, 5 min). Organic extracts were combined and evaporated under a stream of nitrogen. Afterward, the derivatization reaction was performed with the use of DAPTAD as a derivatization agent, for 30 min at room temperature. The mixture was evaporated under the stream of nitrogen and the residue was dissolved in methanol/water (1:1) solution. Finally, 20 μL of an aliquot was subjected to LC-MS/MS analysis.

### 2.5. Data Analysis

The total 25(OH)D concentration was calculated as the sum of 25(OH)D_3_ and 25(OH)D_2_ concentration. Subjects in the study and control groups were classified according to 25(OH)D concentration as severe deficient (≤10 ng/mL), deficient (10–20 ng/mL), insufficient (20–30 ng/mL), and sufficient (>30 ng/mL) [30,31].

### 2.6. Statistical Analyses

Statistical analyses were performed using Statistica 13.3 software (StatSoft, Tulsa, OK, USA). The level of significance was set at *p* < 0.05. The Shapiro–Wilk test was used to determine the distribution of the data. When the data were not distributed normally, nonparametric tests were applied for statistical analyses. For normally distributed data Student’s *t*-test was used. The chi-square test was applied to the categorical variables. The ANOVA Friedman test and post hoc test were used to assess the differences between the concentrations of 25(OH)D_2_, 25(OH)D_3_, 25(OH)D, 24,25(OH)_2_D_3,_ and 3-epi-25(OH)D_3_ in the BC patients prior and after CTH. Patients with missing values were not considered in the statistical analysis for matched pairs. The correlation coefficients were calculated using the Spearman rank correlation test. For Kaplan–Meier analysis, the time from sample collection till the end of the follow-up or till death was taken into account. 

## 3. Results

### 3.1. Vitamin D Status in BC Patients before and during Chemotherapy

In the BC patients group, at baseline, 56.1% of the patients were VD-deficient, 23.5% were insufficient and 20.4% of the patients were sufficient. In the control group, 37.2% of the patients were deficient, 46.5% of the patients were insufficient and 16.3% of the patients were sufficient (Table 2). The proportion of BC patients with 25(OH)D deficiency at baseline was significantly lower than in controls. After 6 months, the number of BC patients with 25(OH)D deficiency increased further to 60% (Table 2). 

### 3.2. Changes in 25(OH)D, 25(OH)D_2_, 25(OH)D_3_, 24,25(OH)_2_D_3_ and 3-epi-25(OH)D_3_ Levels during Chemotherapy

**Figure 1:**
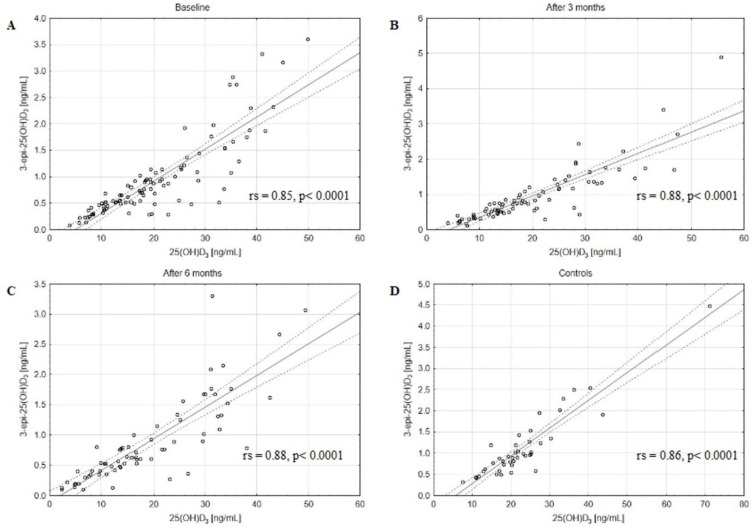
The positive correlations between 3-epi-25(OH)D3 and 25(OH)D3 levels in the BC patients at baseline (**A**), after 3 (**B**) and 6 (**C**) months of CTH, and in the control group (**D**); rs—Spearman’s rank correlation coefficient, solid line represents the regression line, dashed lines represent the 95% CI.

**Figure 2:**
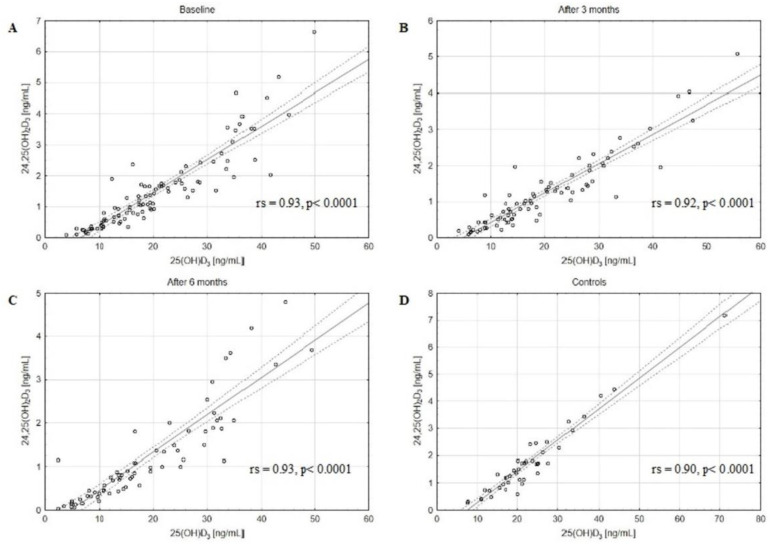
The positive correlations between 24,25(OH)_2_D_3_ and 25(OH)D_3_ levels in the BC patients at baseline (**A**), after 3 (**B**) and 6 (**C**) months of CTH, and in the control group (**D**); rs—Spearman’s rank correlation coefficient, solid line represents the regression line, dashed lines represent the 95% CI.

**Figure 3:**
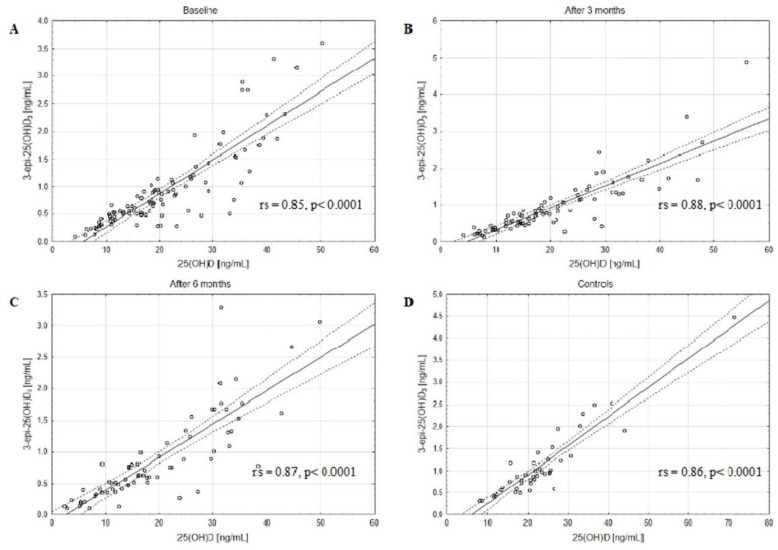
The positive correlations between 3-epi-25(OH)D_3_ and 25(OH)D levels in the BC patients at baseline (**A**), after 3 (**B**) and 6 (**C**) months of CTH, and in the control group (**D**); rs—Spearman’s rank correlation coefficient, solid line represents the regression line, dashed lines represent the 95% CI.

**Figure 4:**
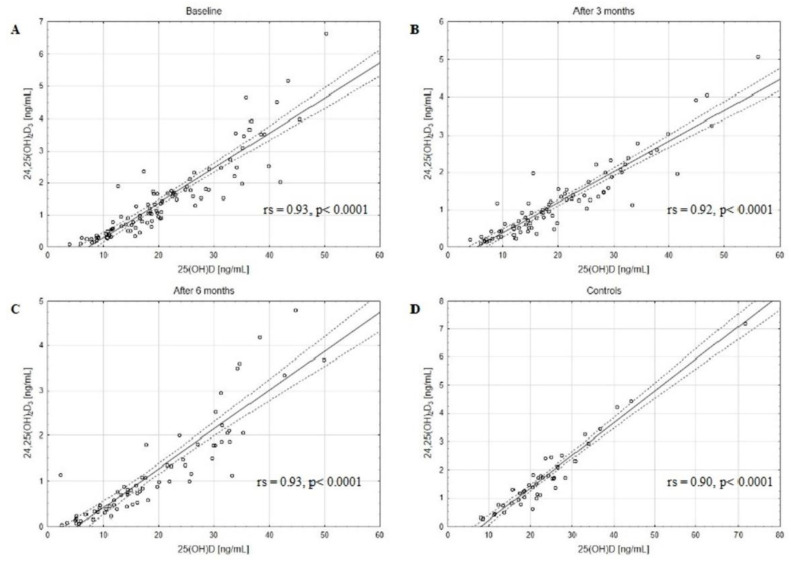
The positive correlations between 24,25(OH)_2_D_3_ and 25(OH)D levels in the BC patients at baseline (**A**), after 3 (**B**) and 6 (**C**) months of CTH and in the control group (**D**); rs—Spearman’s rank correlation coefficient, solid line represents the regression line, dashed lines represent the 95% CI.

The concentrations of assessed VD active forms, 25(OH)D_2_, 25(OH)D_3_, and 25(OH)D, as well as catabolites, 24,25(OH)_2_D_3_ and 3-epi-25(OH)D_3,_ decreased in BC patients after 3 and 6 months of CTH compared to the baseline (Table 2). The matched pairs analysis showed a significant decrease in 25(OH)D, 25(OH)D_2_, 25(OH)D_3_, 24,25(OH)_2_D_3_, and 3-epi-25(OH)D_3_ levels in patients after 3 and 6 months of follow-up (Figure S1). Moreover, a strong positive correlation between the serum concentrations of 3-epi-25(OH)D_3_ and 25(OH)D_3_ in the BC patients at baseline, after 3 and 6 months of CTH, and in the control group was revealed (Figure 1). Similar positive correlations were also observed between 24,25(OH)_2_D_3_ and 25(OH)D_3_ levels (Figure 2). Of note, strong positive correlations were found also between 3-epi-25(OH)D3 and 25(OH)D (Figure 3) as well as 24,25(OH)_2_D_3_ and 25(OH)D levels (Figure 4).

In this study, BC patients receiving neoadjuvant, adjuvant, and palliative CTH, BC patients with different biological subtypes, histological subtypes, clinical stages, BC surgery, radiation therapy, hormone therapy, immunotherapy, and biological subtype were shown to have no significant differences in 25(OH)D, 3-epi-25(OH)D_3,_ and 24,25(OH)_2_D_3_ levels at baseline, after 3, and 6 months of CTH (Kruskal–Wallis test or Mann–Whitney U test, *p* > 0.05).

### 3.3. Predictive Role of Vitamin D Status

**Figure 5:**
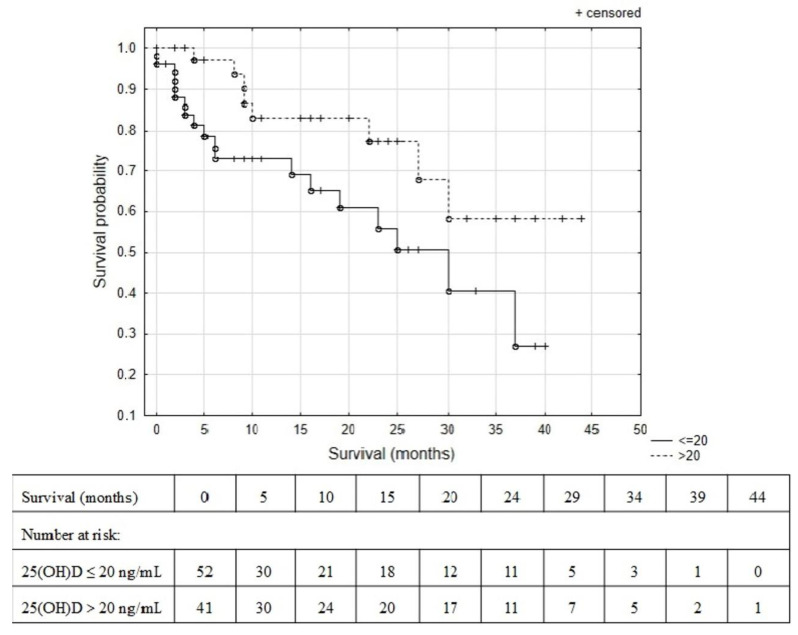
Kaplan–Meier curves for survival probability of BC patients with 25(OH)D levels (a sum of serum levels of 25(OH)D_2_ and 25(OH)D_3_] ≤ 20 ng/mL and > 20 ng/mL at baseline (log-rank test, HR = 2.44 (95% CI 1.07–5.59) *p* = 0.026).

The survival probability of BC patients was analyzed using the Kaplan–Meier estimate for baseline measurement of 25(OH)D levels (Table 1). There was a significant survival difference between BC patients with 25(OH)D levels ≤ 20 ng/mL and >20 ng/mL at baseline (log-rank test, HR = 2.44 (95% CI 1.07–5.59), *p* = 0.026), see Figure 5. The 25th percentile of the survival time was 6 months for BC patients with 25(OH)D level ≤ 20 ng/mL and 23 months for BC patients with 25(OH)D level > 20 ng/mL at baseline. Moreover, the median overall survival was 25 months for patients with 25(OH)D deficiency.

## 4. Discussion

### 4.1. Vitamin D Status in BC Patients at Baseline and during CTH

The aim of this study was to assess baseline VD status [defined as a sum of serum levels of 25-(OH)D_2_ and 25-(OH)D_3_] and changes during CTH using LC–MS/MS methodology in non-supplemented BC patients.

At baseline, 56.1% BC patients were VD-deficient (<20 ng/mL), 23.5% were insufficient and only 20.4% had sufficient VD levels (>30 ng/mL). In comparison with the control group, we found more patients with VD deficiency (56.1% vs. 37.2%) among the BC patients. Decreased levels of VD in BC patients compared to control subjects were observed previously [32,33,34,35,36,37,38,39], and our results confirm these findings with the LC–MS/MS methodology. Presented findings could be a result of decreased sun exposure. However, we cannot exclude the increased demand for VD during carcinogenesis among the BC patients.

Moreover, the concentrations of VD [measured as a sum of 25(OH)D_2_ and 25(OH)D_3_], were further decreased in BC patients after 3 or 6 months of CTH, reaching 60% of BC patients with deficiency after 6 months. In an Indonesian study, similar results were recently published with the ELISA methodology. Severe VD deficiency [25(OH)D_2_ and 25(OH)D_3_) was found in 82.4% of BC patients at baseline and the rate further increased to 89.0% after CTH [16]. Kok et al. recorded decreased level of 25(OH)D_3_ 1 to 3 weeks after CTH compared to 25(OH)D_3_ status prior to CTH in BC patients using the LC-MS/MS method; however only one VD form was assessed [33]. Kim et al. showed that only 26.9% of BC women were VD-sufficient after 6 months of CTH compared to 49.5% before CTH [40]. Our observations provide further evidence regarding the necessity of VD assessments and supplementation during CTH in BC patients. It seems even more important in the context of recent findings by El-Bassiouny et al. showing promising clinical evidence to support the cardioprotective effects of VD against pro-inflammatory cytokines induced by doxorubicin CTH [41].

### 4.2. Catabolites Status of 24,25(OH)_2_D_3_ and 3-epi-25(OH)D_3_ at Baseline and during Chemotherapy

Another aim of this study was to determine the level of VD catabolites, 24,25(OH)_2_D_3_ and 3-epi-25-(OH)D_3_, to provide further data regarding VD catabolic pathways in BC patients.

We found that the concentrations of the main catabolites, 24,25(OH)_2_D_3_ and 3-epi-25(OH)D_3_, were decreased at baseline in comparison to the control group and decreased in BC patients after 3 and 6 months of CTH as compared to baseline. Strong positive correlations between the serum concentrations of 3-epi-25(OH)D_3_ and 25(OH)D in the BC patients at baseline, after 3 and 6 months of CTH, and in the control group were found. Similar correlations were also observed between 24,25(OH)_2_D_3_ and 25(OH)D levels. Additionally, we revealed strong positive correlations between 3-epi-25(OH)D_3_ and 25(OH)D_3_, 24,25(OH)_2_D_3,_ and 25(OH)D_3_ levels both in the study and control groups. The clinical implication of the observed strong correlations could be the limitation of VD assessment to only 25(OH)D levels. However, the results should be confirmed in other studies.

The presented results provide evidence that both catabolic pathways, via 24-hydroxylase enzyme into 24,25(OH)_2_D and 1,24,25(OH)_3_D, considered as inert metabolites, and via C3-epimerase catabolic pathway [22,23] resulting in 3-epi-25(OH)D_3_ synthesis, are engaged in the catabolism of VD in BC patients, similar to healthy subjects.

Although several authors showed the positive correlation between serum concentrations of 3-epi-25(OH)D_3_ and 25(OH)D_3_ using the LC-MS/MS method [42,43,44] in different populations, no data so far has been published for BC patients. To our knowledge, this is the first paper evaluating the impact of CTH on 24,25(OH)_2_D_3_ and 3-epi-25(OH)D_3_ metabolites in BC patients.

### 4.3. Predictive Role of Vitamin D Status

The additional goal of this study was to evaluate the predictive role of VD status in Caucasian BC patients treated with CTH. The Kaplan–Meier survival analysis showed poorer survival probability in BC patients with 25(OH)D levels ≤ 20 ng/mL at baseline.

Previously published results were inconsistent, but many authors observed an inverse association between VD levels before treatment and mortality in BC patients [45,46]; others did not confirm this relationship [47]. Among others, Tokunaga et al. showed that levels of VD over 23.6 ng/mL diminished the risk of BC-related mortality in newly diagnosed patients [9]. Vrieling et al. in a prospective study of BC patients found that a lower level of 25(OH)D assessed via enzyme immunoassay methodology was related to a higher risk of death [48]. In another study, Yao et al. investigated the predictive role of 25(OH)D serum levels measured at the time of diagnosis and found that 25(OH)D concentrations measured via immunochemiluminometric assay were inversely associated with the risk of disease progression and death [49].

Such results seem to justify vitamin VD supplementation in BC patients. Several studies analyzed the supplementation of VD post cancer diagnosis. A recent study showed that VD supplementation was associated with lower total mortality [50]. Other authors found that de novo post-diagnostic supplementation of VD was associated with a 20% reduction in breast cancer-specific mortality in a large cohort study [51].

The LC-MS/MS methodology presents a possibility to reliably assess VD and spectrum of its metabolites. The main novelty of this study is presenting VD catabolism data in BC patients using the LC-MS/MS methodology. Altered VD catabolism via 24-hydroxylase and 3-epimerase pathways could potentially contribute to decreased VD levels in BC patients. We showed that that VD catabolism is not affected in Caucasian BC patients receiving CHT. Correlations observed between VD and its catabolites allow for assessment of only 25(OH)D in the Caucasian BC patients. Moreover, this study provides additional data regarding the negative impact of VD deficiency for OS in Caucasian BC patients.

We realize some of this study’s limitations, including a lack of seasonal analysis related to sunshine exposure, assessment of eating habits, and heterogeneity of the group of BC patients starting CTH. Moreover, due to splitting the patients into deficiency/non-deficiency subgroups, the sample size was smaller, which can affect the statistical analysis.

## 5. Conclusions

The presented data provide further evidence that BC patients before CTH are more VD-deficient than the general population, and this deficiency increases further during the CTH treatment, as observed using the reference LC-MS/MS methodology. VD metabolites produced via 24-hydroxylase and C3-epimerase pathways could potentially contribute to VD deficiency. They show that VD catabolism is not affected in BC patients.

VD is considered an important factor in BC development, affecting BC cells metabolism and proliferation. VD deficiency can impact overall survival in BC patients’ population. The presented results confirm decreased survival in VD-deficient BC patients. This finding could suggest that VD supplementation is beneficial for patients with 25(OH)D levels below 20 ng/mL. However, the benefits from supplementation may not translate directly into improved survival, and such recommendations should be assessed in the future studies.

## Figures and Tables

**Table 1:** Characteristics of the study group with stratification according to Vitamin D status.

Parameter	BC Patients N = 98	25(OH)D ≤ 20 ng/mL N = 55	25(OH)D > 20 ng/mL N = 43	*p*-Value ^4^
Age [years]	58.4 ± 11.0	58.1 ± 12.0	58.9 ± 9.8	0.72 ^1^
Body mass index [kg/m^2^]	26.7 (24.5–29.9)	26.4 (24.3–30.1)	27.0 (24.6–29.9)	0.77 ^2^
Clinical stage:				0.06 ^3^
I	14 (14%)	11 (20%)	3 (7%)	
II	44 (45%)	18 (33%)	26 (60%)	
III	29 (30%)	18 (33%)	11 (26%)	
IV	8 (8%)	5 (9%)	3 (7%)	
n/a	2 (2%)	2 (4%)	0 (0%)	
Histological subtype:				0.30 ^3^
NST	74 (76%)	41 (76%)	33 (77%)	
lobular	17 (18%)	8 (15%)	9 (21%)	
others	6 (6%)	5 (9%)	1 (2%)	
Biological subtype:				0.43 ^3^
luminal A	19 (20%)	7 (13%)	12 (28%)	
luminal B	33 (34%)	20 (37%)	13 (30%)	
luminal B HER2 positive	16 (16%)	9 (17%)	7 (16%)	
non-luminal HER2 positive	13 (13%)	8 (15%)	5 (12%)	
triple negative	16 (16%)	10 (19%)	6 (14%)	
Chemotherapy:				0.25 ^3^
neoadjuvant	23 (24%)	12 (23%)	11 (26%)	
adjuvant	41 (43%)	20 (38%)	21 (50%)	
palliative	31 (33%)	21 (40%)	10 (24%)	

Data presented as mean values ± SD or median (1–3Q). ^1^ Student’s *t*-test; ^2^ Mann–Whitney U test; ^3^ chi-square test; ^4^ Student’s *t*-test, Mann–Whitney U test, chi-square test analyses performed for comparison between 25(OH)D ≤ 20 ng/mL and 25(OH)D > 20 ng/mL groups; NST—invasive carcinoma of no special type.

**Table 2:** Results of 25(OH)D status (sum of 25(OH)D_2_ and 25(OH)D_3_ concentrations), 25(OH)D_2_, 25(OH)D_3_, 24,25(OH)_2_D_3_ and 3-epi-25(OH)D_3_ serum levels in the BC patients at baseline and after 3 and 6 months of CTH compared to the control group. Kruskall–Wallis test was non-significant for comparison among all tested groups (control group, baseline, 3 months, 6 months, (*p* > 0.05). *p* values were calculated for each group: baseline, after 3 and 6 months vs. control group. Chi-square test was significant for comparisons among each group (baseline, 3 and 6 months) vs. the control group.

Vitamin D Status (25(OH)D [ng/mL])	Control Group N (%)	Breast Cancer Patient Group N (%)					
		Baseline	*p*-Value ^1^	after 3 Months	*p*-Value ^1^	after 6 Months	*p*-Value ^1^
Severe deficiency (≤10)	2 (4.7%)	12 (12.2%)	0.04	15 (18.5%)	0.03	15 (21.4%)	0.002
Deficiency (10–20)	14 (32.5%)	43 (43.9%)		33 (40.7%)		27 (38.6%)	
Insufficiency (20–30)	20 (46.5%)	23 (23.5%)		19 (23.5%)		11 (15.7%)	
Sufficiency (>30)	7 (16.3%)	20 (20.4%)		14 (17.3%)		17 (24.3%)	
**Vitamin D and Vitamin D Metabolites [ng/mL] ^2^**	**Control Group**	**Breast Cancer Patient Group**					
		**Baseline**		**after 3 Months**		**after 6 Months**	
25(OH)D	21.45 (17.23–25.94)	18.91 (13.07–26.60)		17.85 (12.66–27.11)		16.63 (11.00–29.91)	
25(OH)D_2_	0.44 (0.29–0.70)	0.44 (0.29–0.71)		0.42 (0.27–0.63)		0.38 (0.26–0.59)	
25(OH)D_3_	21.08 (16.90–25.10)	18.31 (12.69–26.34)		17.39 (12.23–26.92)		16.30 (9.84–29.64)	
24,25(OH)_2_D_3_	1.47 (0.96–2.12)	1.18 (0.57–1.88)		1.02 (0.51–1.56)		0.89 (0.44–1.81)	
3-epi-25(OH)D_3_	0.92 (0.59–1.27)	0.71 (0.48–1.14)		0.74 (0.45–1.29)		0.65 (0.40–1.25)	

^1^ chi-square test, ^2^ data presented as medians with 25th and 75th percentiles.

## Data Availability

The data presented in this study are available on request from the corresponding author. The data are not publicly available due to subject privacy restrictions.

## Supplementary Materials

The following supporting information can be downloaded at: https://www.mdpi.com/article/10.3390/metabo13090996/s1, Figure S1. The quantitative relationships between serum levels of 25(OH)D_2_ (A), 25(OH)D_3_ (B), 25(OH)D (C), 24,25(OH)_2_D_3_ (D) and 3-epi-25(OH)D_3_ (E) in the paired samples of the breast cancer patients at baseline and after chemotherapy. Each line represents an individual subject, the bold black lines represent the median values, * *p* < 0.05 ANOVA Friedman test followed with post hoc test, N = 68.
