# Supplementary material for: Vitamin D and Its Metabolites Status before and during Chemotherapy in Caucasian Breast Cancer Patients
Source: Metabolites. 2023 Sep 6;13(9):996. doi: 10.3390/metabo13090996 (PMC10534610; doi:10.3390/metabo13090996)
Supplement: Supplementary file 1 [file metabolites-13-00996-s001.zip › metabolites-2540381-supplementary.pdf]

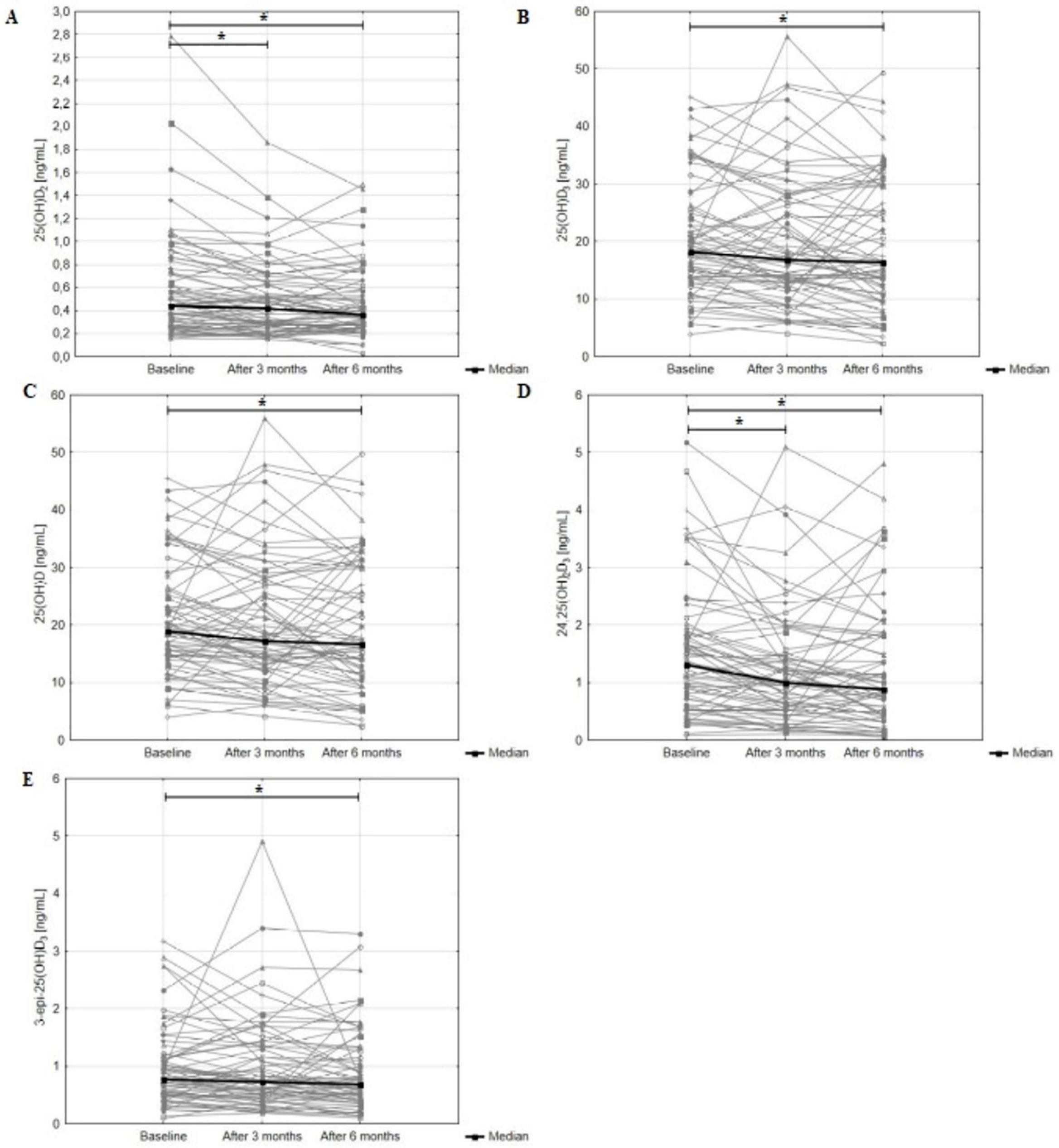

Figure S1. The quantitative relationships between serum levels of $25(OH)D_2$ (**A**), $25(OH)D_3$ (**B**), 25(OH)D (**C**), $24,25(OH)_2D_3$ (**D**) and 3-epi-$25(OH)D_3$ (**E**) in the paired samples of the breast cancer patients at baseline and after chemotherapy. Each line represents an individual subject, the bold black lines represent the median values, * $p < 0.05$ ANOVA Friedman test followed with post hoc test, N = 68.
